# Crystal structure of bis­[(acetato-κ*O*)(imidazolidine-2-thione-κ*S*)bis­(tri­phenyl­phosphane-κ*P*)silver(I)] di-μ-imidazol­idine-2-thione-κ^4^ *S*:*S*-bis­[(imidazol­id­ine-2-thione-κ*S*)bis­(tri­phenyl­phosphane-κ*P*)silver(I)] di­acetate aceto­nitrile disolvate tetra­hydrate

**DOI:** 10.1107/S205698901600308X

**Published:** 2016-03-04

**Authors:** Arunpatcha Nimthong-Roldán, Janejira Ratthiwan, Sawanya Lakmas, Yupa Wattanakanjana

**Affiliations:** a Department of Chemistry, Youngstown State University, 1 University Plaza, 44555 Youngstown, OH, USA; b Department of Chemistry, Faculty of Science, Prince of Songkla University, Hat Yai, Songkhla 90112, Thailand

**Keywords:** crystal structure, imidazolidine-2-thione, silver complex

## Abstract

In the title compound, [Ag_2_(C_3_H_6_N_2_S)_4_(C_18_H_15_P)_2_](C_2_H_3_O_2_)_2_·[Ag(C_2_H_3_O_2_)(C_3_H_6_N_2_S)(C_18_H_15_P)_2_]_2_·2C_2_H_3_N·4H_2_O, the Ag^I^ ion in the mononuclear neutral complex exhibits a distorted tetra­hedral environment with coordination by two P atoms from tri­phenyl­phosphane (PPh_3_) ligands, one S atom of an imidazolidine-2-thione (etu) ligand and one O atom of an acetate anion. The binuclear cationic complex comprises two inversion-related [Ag(C_3_H_6_N_2_S)_2_(C_18_H_15_P)] units with Ag^I^ ions bridged by two S atoms from etu ligands forming a four-membered Ag–S–Ag–S ring. Each Ag^I^ ion is coordinated by a P atom of a PPh_3_ ligand, two S atoms of bridging etu ligands and the terminal S atom of an etu ligand in a distorted tetra­hedral environment. In the crystal, the mononuclear complex is linked to lattice water mol­ecules through O—H⋯O and N—H⋯O hydrogen bonds, forming a chain along [100]. In addition, the binuclear complex mol­ecules are connected to acetate anions and lattice water mol­ecules *via* O—H⋯O, N—H⋯O and O—H⋯S hydrogen bonds, also along [100].

## Abstract 2

The title compound consists of a mononuclear Ag^I^ complex, a discrete binuclear Ag^I^ complex, acetate anions, aceto­nitrile solvent mol­ecules and water mol­ecules. The mol­ecular components are linked through O—H⋯O, N—H⋯O and O—H⋯S hydrogen bonds, forming a chain structure along [100].

## Chemical context   

Silver(I) complexes containing S- or P-donor ligands have received much attention because of their potential applications in biochemistry (Isab *et al.*, 2010 ▸; Nawaz *et al.*, 2011 ▸) and their luminescent properties (Ferrari *et al.*, 2007 ▸). The crystal structure of the title compound, [Ag(CH_3_COO)(C_3_H_6_N_2_S)(C_18_H_15_P)_2_]_2_·[Ag_2_(C_3_H_6_N_2_S)_4_(C_18_H_15_P)_2_](CH_3_COO)_2_·2CH_3_CN·4H_2_O, is presented herein.
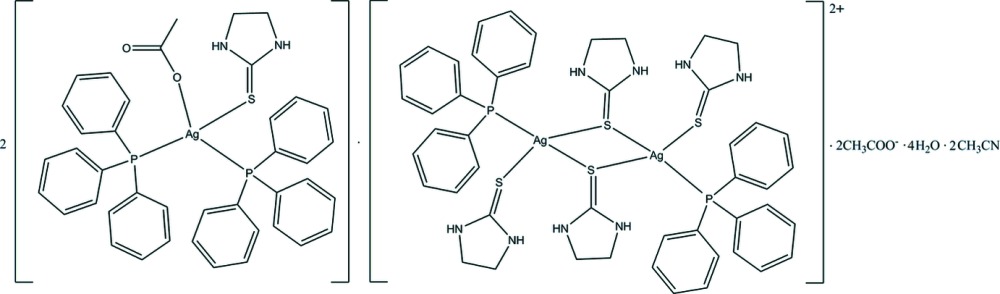


## Structural commentary   

**Figure 1:**
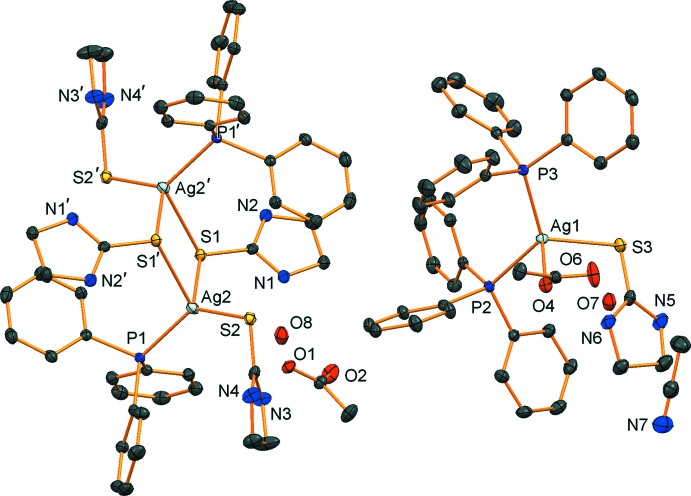
The structures of the mol­ecular components with displacement ellipsoids drawn at the 50% probability level. H atoms are omitted for clarity. The symmetry operator for equivalent atoms of the discrete dimer is (−*x*, −*y*, −*z* + 1). Only the symmetry-unique mononuclear complex, acetate ion, aceto­nitrile solvent mol­ecule and solvent water mol­ecules are shown.

The structures of the mol­ecular components of the title compound are shown in Fig. 1 ▸. The crystal structure consists of a mononuclear neutral Ag^I^ complex, a discrete cationic binuclear Ag^I^ complex, acetate anions, aceto­nitrile solvent mol­ecules and water mol­ecules of crystallization. In the mononuclear complex, the Ag^I^ ion exhibits a distorted tetra­hedral environment, being coordinated by two P atoms of tri­phenyl­phosphane (PPh_3_) ligands, one terminal S atom of a imidazolidine-2-thione (or ethyl­ene­thio­urea; etu) ligand and an O atom of an acetate anion. The mol­ecule of the binuclear complex lies across a crystallographic inversion center which is at the center of the Ag_2_S_2_ core. The two Ag^I^ ions are bridged by two S atoms of etu ligands forming a four-membered Ag–S–Ag–S ring. Each Ag^I^ ion is in a distorted tetra­hedral coordination geometry formed by a P atom of a PPh_3_ ligand, the S atoms of two bridging etu ligands and a terminal S atom of an etu ligand.

## Supra­molecular features   

**Figure 2:**
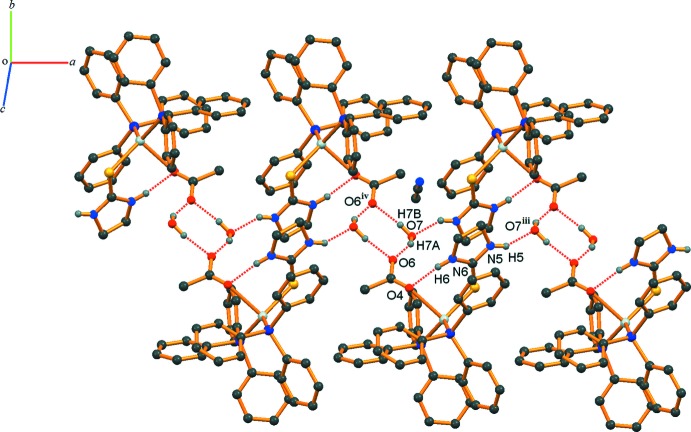
Part of the crystal structure with O—H⋯O and N—H⋯O hydrogen bonds shown as dashed lines (symmetry codes as in Table 1 ▸).

**Figure 3:**
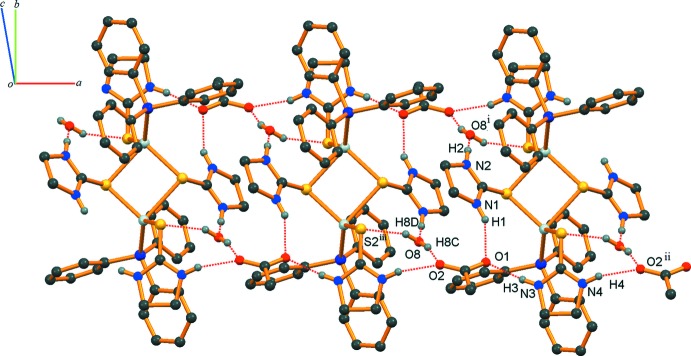
Part of the crystal structure with O—H⋯O, N—H⋯O and O—H⋯S hydrogen bonds shown as dashed lines (symmetry codes as in Table 1 ▸).

In the crystal structure, O7—H7*A*⋯O6, O7—H7*B*⋯O6^iv^, N6—H6⋯O4 and N5—H5⋯O7^iii^ hydrogen bonds (symmetry codes as in Table 1 ▸) link the mononuclear complexes and water mol­ecules into chains along [100] (Fig. 2 ▸). In addition, N1—H1⋯O1, N2—H2⋯O8^i^, N3—H3⋯O1, N4—H4⋯O2^ii^, O8—H8*C*⋯O2 and O8—H8*D*⋯S2^iii^ hydrogen bonds connect the discrete binuclear complexes with acetate anions and water mol­ecules, forming another chain along [100] (Fig. 3 ▸).

## Synthesis and crystallization   

Tri­phenyl­phosphane (0.31 g, 1.18 mmol) was dissolved in 30 cm^3^ of aceto­nitrile at 335 K. Ag(OAc) (0.10 g, 0.60 mmol) was added and the mixture was stirred for 3 h. Ethyl­ene­thio­urea (0.06 g, 0.59 mmol) was added and the new reaction mixture was heated under reflux for 5 h. The resulting clear solution was filtered off and left to evaporate at room temperature. Crystals suitable for X-ray diffraction, which were deposited upon standing for a week, were filtered off and dried under reduced pressure.

## Refinement   

Crystal data, data collection and structure refinement details are summarized in Table 2 ▸. All C- and N-bound H atoms were constrained with a riding model: 0.95 Å (aryl H) and *U*_iso_(H) = 1.2*U*_eq_(C); 0.99 Å (CH_2_) and *U*_iso_(H) = 1.2*U*_eq_(C); 0.98 Å (CH_3_) and *U*_iso_(H) = 1.5*U*_eq_(C); 0.88 Å (NH) and *U*_iso_(H) = 1.2*U*_eq_(N). Water hydrogen atoms were located from a difference Fourier map and were refined with an O—H distance restraint of 0.84 (2) Å and *U*_iso_(H) = 1.2*U*_eq_(O).

## Supplementary Material

Crystal structure: contains datablock(s) I, New_Global_Publ_Block. DOI: 10.1107/S205698901600308X/wm5275sup1.cif

Structure factors: contains datablock(s) I. DOI: 10.1107/S205698901600308X/wm5275Isup2.hkl

CCDC reference: 1452047

Additional supporting information:  crystallographic information; 3D view; checkCIF report

## Figures and Tables

**Table 1:** Hydrogen-bond geometry (Å, °)

*D*—H⋯*A*	*D*—H	H⋯*A*	*D*⋯*A*	*D*—H⋯*A*
N1—H1⋯O1	0.88	2.10	2.908 (3)	153
N2—H2⋯O8^i^	0.88	2.00	2.808 (3)	152
N3—H3⋯O1	0.88	1.94	2.780 (3)	160
N4—H4⋯O2^ii^	0.88	2.09	2.864 (3)	146
N5—H5⋯O7^iii^	0.88	1.96	2.767 (3)	152
N6—H6⋯O4	0.88	1.91	2.780 (3)	172
O7—H7*A*⋯O6	0.85 (1)	1.88 (1)	2.716 (3)	169 (4)
O7—H7*B*⋯O6^iv^	0.84 (1)	2.00 (2)	2.782 (3)	154 (3)
O8—H8*C*⋯O2	0.84 (1)	1.88 (1)	2.709 (3)	167 (3)
O8—H8*D*⋯S2^iii^	0.84 (1)	2.56 (2)	3.357 (2)	159 (3)

Symmetry codes: (i) 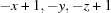; (ii) 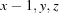; (iii) 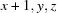; (iv) 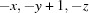.

**Table 2:** Experimental details

Crystal data	
Chemical formula	[Ag_2_(C_3_H_6_N_2_S)_4_(C_18_H_15_P)_2_](C_2_H_3_O_2_)_2_·[Ag(C_2_H_3_O_2_)(C_3_H_6_N_2_S)(C_18_H_15_P)_2_]_2_·2C_2_H_3_N·4H_2_O
*M*_r_	3008.39
Crystal system, space group	Triclinic, *P* 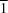
Temperature (K)	100
*a*, *b*, *c* (Å)	9.5867 (4), 16.3018 (7), 22.8548 (10)
α, β, γ (°)	104.179 (2), 94.449 (2), 91.992 (2)
*V* (Å^3^)	3447.2 (3)
*Z*	1
Radiation type	Mo *K*α
μ (mm^−1^)	0.78
Crystal size (mm)	0.16 × 0.15 × 0.13

Data collection	
Diffractometer	Bruker AXS D8 Quest CMOS
Absorption correction	Multi-scan (*SADABS*; Bruker, 2014 ▸)
*T*_min_, *T*_max_	0.882, 0.903
No. of measured, independent and observed [*I* > 2σ(*I*)] reflections	71762, 20957, 18898
*R*_int_	0.026
(sin θ/λ)_max_ (Å^−1^)	0.716

Refinement	
*R*[*F* ^2^ > 2σ(*F* ^2^)], *wR*(*F* ^2^), *S*	0.042, 0.088, 1.26
No. of reflections	20957
No. of parameters	826
No. of restraints	6
H-atom treatment	H atoms treated by a mixture of independent and constrained refinement
Δρ_max_, Δρ_min_ (e Å^−3^)	1.05, −0.97

Computer programs: *APEX2* and *SAINT* (Bruker, 2014 ▸), *SHELXS97* (Sheldrick, 2008 ▸), *SHELXL2014* (Sheldrick, 2015 ▸), *SHELXLE* (Hübschle *et al.*, 2011 ▸), *Mercury* (Macrae *et al.*, 2008 ▸) and *publCIF* (Westrip, 2010 ▸).

## supplementary crystallographic information

### Crystal data

**Table:**

[Ag_2_(C_3_H_6_N_2_S)_4_(C_18_H_15_P)_2_](C_2_H_3_O_2_)_2_·[Ag(C_2_H_3_O_2_)(C_3_H_6_N_2_S)(C_18_H_15_P)_2_]_2_·2C_2_H_3_N·4H_2_O	*Z* = 1
*M**_r_* = 3008.39	*F*(000) = 1548
Triclinic, *P*1	*D*_x_ = 1.449 Mg m^−^^3^
*a* = 9.5867 (4) Å	Mo *K*α radiation, λ = 0.71073 Å
*b* = 16.3018 (7) Å	Cell parameters from 9707 reflections
*c* = 22.8548 (10) Å	θ = 2.4–30.6°
α = 104.179 (2)°	µ = 0.78 mm^−^^1^
β = 94.449 (2)°	*T* = 100 K
γ = 91.992 (2)°	Block, colourless
*V* = 3447.2 (3) Å^3^	0.16 × 0.15 × 0.13 mm

### Data collection

**Table:**

Bruker AXS D8 Quest CMOS diffractometer	20957 independent reflections
Radiation source: I-mu-S microsource X-ray tube	18898 reflections with *I* > 2σ(*I*)
Laterally graded multilayer (Goebel) mirror monochromator	*R*_int_ = 0.026
ω and φ scans	θ_max_ = 30.6°, θ_min_ = 2.3°
Absorption correction: multi-scan (*SADABS*; Bruker, 2014)	*h* = −13→13
*T*_min_ = 0.882, *T*_max_ = 0.903	*k* = −23→23
71762 measured reflections	*l* = −32→32

### Refinement

**Table:**

Refinement on *F*^2^	Primary atom site location: structure-invariant direct methods
Least-squares matrix: full	Secondary atom site location: difference Fourier map
*R*[*F*^2^ > 2σ(*F*^2^)] = 0.042	Hydrogen site location: mixed
*wR*(*F*^2^) = 0.088	H atoms treated by a mixture of independent and constrained refinement
*S* = 1.26	*w* = 1/[σ^2^(*F*_o_^2^) + (0.0001*P*)^2^ + 7.6382*P*] where *P* = (*F*_o_^2^ + 2*F*_c_^2^)/3
20957 reflections	(Δ/σ)_max_ = 0.002
826 parameters	Δρ_max_ = 1.05 e Å^−^^3^
6 restraints	Δρ_min_ = −0.97 e Å^−^^3^

### Special details

**Table:**

Geometry. All e.s.d.'s (except the e.s.d. in the dihedral angle between two l.s. planes) are estimated using the full covariance matrix. The cell e.s.d.'s are taken into account individually in the estimation of e.s.d.'s in distances, angles and torsion angles; correlations between e.s.d.'s in cell parameters are only used when they are defined by crystal symmetry. An approximate (isotropic) treatment of cell e.s.d.'s is used for estimating e.s.d.'s involving l.s. planes.

### Fractional atomic coordinates and isotropic or equivalent isotropic displacement parameters (Å^2^)

**Table:**

	*x*	*y*	*z*	*U*_iso_*/*U*_eq_	
Ag1	0.35807 (2)	0.29775 (2)	0.13070 (2)	0.01524 (4)	
Ag2	0.00978 (2)	0.12147 (2)	0.53974 (2)	0.01655 (4)	
S1	0.19225 (6)	0.01029 (4)	0.50712 (3)	0.01504 (10)	
S2	−0.08966 (6)	0.20987 (4)	0.47189 (3)	0.01634 (11)	
S3	0.51845 (7)	0.36676 (4)	0.07081 (3)	0.02254 (13)	
P1	0.05184 (6)	0.15244 (4)	0.64996 (3)	0.01168 (10)	
P2	0.41455 (6)	0.32402 (4)	0.24144 (3)	0.01230 (10)	
P3	0.21550 (6)	0.17470 (4)	0.07067 (3)	0.01362 (11)	
N1	0.3296 (2)	0.11367 (13)	0.45141 (10)	0.0187 (4)	
H1	0.2904	0.1591	0.4711	0.022*	
N2	0.3752 (2)	−0.01974 (14)	0.42198 (10)	0.0212 (4)	
H2	0.3663	−0.0748	0.4178	0.025*	
N3	0.0389 (2)	0.34962 (14)	0.54878 (11)	0.0213 (4)	
H3	0.1202	0.3263	0.5496	0.026*	
N4	−0.1850 (2)	0.36111 (14)	0.52721 (11)	0.0237 (5)	
H4	−0.2693	0.3491	0.5081	0.028*	
N5	0.6169 (2)	0.53077 (15)	0.10791 (12)	0.0261 (5)	
H5	0.6987	0.5204	0.0935	0.031*	
N6	0.4141 (2)	0.50600 (14)	0.13962 (11)	0.0218 (4)	
H6	0.3336	0.4795	0.1415	0.026*	
C1	0.3025 (2)	0.03624 (14)	0.45799 (10)	0.0140 (4)	
C2	0.4331 (3)	0.11450 (16)	0.40730 (11)	0.0200 (5)	
H2A	0.5159	0.1527	0.4258	0.024*	
H2B	0.3917	0.1322	0.3716	0.024*	
C3	0.4719 (3)	0.02121 (17)	0.39010 (12)	0.0213 (5)	
H3A	0.4573	−0.0029	0.3458	0.026*	
H3B	0.5706	0.0154	0.4041	0.026*	
C4	−0.0783 (2)	0.31014 (15)	0.51795 (11)	0.0161 (4)	
C5	0.0153 (3)	0.43661 (19)	0.58100 (17)	0.0362 (8)	
H5A	0.0616	0.4782	0.5626	0.043*	
H5B	0.0499	0.4480	0.6243	0.043*	
C6	−0.1436 (3)	0.43931 (17)	0.57316 (14)	0.0265 (6)	
H6A	−0.1855	0.4400	0.6115	0.032*	
H6B	−0.1717	0.4896	0.5591	0.032*	
C7	0.5159 (3)	0.47123 (16)	0.10718 (12)	0.0190 (5)	
N7	0.2420 (3)	0.76608 (18)	0.14911 (13)	0.0381 (6)	
C8	0.5723 (3)	0.61517 (18)	0.13593 (16)	0.0312 (6)	
H8A	0.5381	0.6447	0.1050	0.037*	
H8B	0.6492	0.6504	0.1629	0.037*	
C9	0.4533 (3)	0.59427 (17)	0.17195 (13)	0.0246 (5)	
H9A	0.4864	0.5991	0.2148	0.030*	
H9B	0.3743	0.6313	0.1701	0.030*	
C11	−0.0123 (2)	0.24930 (14)	0.69579 (10)	0.0142 (4)	
C12	0.0554 (3)	0.29479 (15)	0.75139 (11)	0.0180 (4)	
H12	0.1400	0.2756	0.7666	0.022*	
C13	−0.0003 (3)	0.36812 (17)	0.78470 (12)	0.0232 (5)	
H13	0.0460	0.3985	0.8224	0.028*	
C14	−0.1231 (3)	0.39624 (17)	0.76243 (14)	0.0264 (6)	
H14	−0.1606	0.4464	0.7847	0.032*	
C15	−0.1915 (3)	0.35125 (17)	0.70770 (14)	0.0261 (6)	
H15	−0.2767	0.3703	0.6930	0.031*	
C16	−0.1362 (3)	0.27823 (16)	0.67406 (12)	0.0207 (5)	
H16	−0.1831	0.2482	0.6363	0.025*	
C21	0.2363 (2)	0.15765 (14)	0.67815 (10)	0.0136 (4)	
C22	0.2822 (3)	0.13182 (16)	0.72991 (11)	0.0175 (4)	
H22	0.2181	0.1040	0.7494	0.021*	
C23	0.4223 (3)	0.14682 (17)	0.75313 (12)	0.0222 (5)	
H23	0.4528	0.1304	0.7889	0.027*	
C24	0.5172 (3)	0.18550 (17)	0.72422 (12)	0.0229 (5)	
H24	0.6122	0.1965	0.7405	0.027*	
C25	0.4728 (3)	0.20824 (17)	0.67126 (12)	0.0212 (5)	
H25	0.5384	0.2333	0.6508	0.025*	
C26	0.3329 (2)	0.19467 (15)	0.64797 (11)	0.0170 (4)	
H26	0.3032	0.2104	0.6118	0.020*	
C31	−0.0272 (2)	0.06841 (14)	0.67890 (10)	0.0131 (4)	
C30	0.3989 (2)	0.33050 (16)	0.51551 (11)	0.0175 (4)	
C32	−0.1146 (2)	0.08315 (16)	0.72606 (11)	0.0175 (4)	
H32	−0.1337	0.1395	0.7457	0.021*	
C33	−0.1739 (3)	0.01499 (18)	0.74438 (12)	0.0225 (5)	
H33	−0.2336	0.0251	0.7764	0.027*	
C34	−0.1459 (3)	−0.06732 (18)	0.71596 (12)	0.0235 (5)	
H34	−0.1862	−0.1134	0.7287	0.028*	
C35	−0.0590 (3)	−0.08267 (16)	0.66897 (12)	0.0218 (5)	
H35	−0.0397	−0.1392	0.6497	0.026*	
C36	−0.0004 (3)	−0.01517 (15)	0.65027 (11)	0.0172 (4)	
H36	0.0582	−0.0258	0.6179	0.021*	
C41	0.5210 (2)	0.42249 (15)	0.27211 (10)	0.0145 (4)	
C40	0.3705 (3)	0.41859 (18)	0.50781 (15)	0.0291 (6)	
H40A	0.4002	0.4241	0.4688	0.044*	
H40B	0.4229	0.4611	0.5407	0.044*	
H40C	0.2700	0.4273	0.5089	0.044*	
C42	0.6444 (3)	0.43300 (16)	0.24514 (12)	0.0190 (5)	
H42	0.6721	0.3887	0.2135	0.023*	
C43	0.7267 (3)	0.50853 (17)	0.26481 (13)	0.0232 (5)	
H43	0.8117	0.5149	0.2472	0.028*	
C44	0.6852 (3)	0.57443 (17)	0.30988 (13)	0.0251 (5)	
H44	0.7412	0.6259	0.3229	0.030*	
C45	0.5623 (3)	0.56490 (16)	0.33580 (12)	0.0237 (5)	
H45	0.5330	0.6103	0.3662	0.028*	
C46	0.4808 (3)	0.48901 (16)	0.31759 (11)	0.0195 (5)	
H46	0.3973	0.4826	0.3363	0.023*	
C51	0.5182 (2)	0.24246 (15)	0.26282 (10)	0.0141 (4)	
C50	0.0661 (2)	0.41834 (15)	0.11199 (11)	0.0161 (4)	
C52	0.4795 (3)	0.15807 (15)	0.23323 (11)	0.0171 (4)	
H52	0.4037	0.1458	0.2025	0.021*	
C53	0.5502 (3)	0.09187 (17)	0.24805 (12)	0.0217 (5)	
H53	0.5226	0.0348	0.2277	0.026*	
C54	0.6620 (3)	0.10971 (17)	0.29293 (12)	0.0218 (5)	
H54	0.7109	0.0648	0.3032	0.026*	
C55	0.7016 (3)	0.19306 (18)	0.32251 (12)	0.0227 (5)	
H55	0.7778	0.2050	0.3530	0.027*	
C56	0.6304 (3)	0.25949 (17)	0.30779 (11)	0.0195 (5)	
H56	0.6581	0.3164	0.3283	0.023*	
C61	0.2704 (2)	0.33088 (14)	0.29028 (10)	0.0138 (4)	
C60	−0.0750 (3)	0.39096 (18)	0.12854 (13)	0.0230 (5)	
H60A	−0.1242	0.3505	0.0935	0.034*	
H60B	−0.1308	0.4406	0.1405	0.034*	
H60C	−0.0614	0.3641	0.1624	0.034*	
C62	0.2795 (3)	0.30307 (16)	0.34366 (11)	0.0178 (4)	
H62	0.3643	0.2817	0.3566	0.021*	
C63	0.1651 (3)	0.30661 (16)	0.37794 (11)	0.0193 (5)	
H63	0.1717	0.2873	0.4140	0.023*	
C64	0.0407 (3)	0.33849 (16)	0.35943 (11)	0.0199 (5)	
H64	−0.0376	0.3406	0.3827	0.024*	
C65	0.0315 (3)	0.36712 (18)	0.30680 (12)	0.0223 (5)	
H65	−0.0528	0.3897	0.2945	0.027*	
C66	0.1455 (3)	0.36293 (17)	0.27185 (11)	0.0188 (5)	
H66	0.1382	0.3818	0.2356	0.023*	
C71	0.2823 (2)	0.07331 (15)	0.07579 (10)	0.0159 (4)	
C72	0.4256 (3)	0.06359 (19)	0.07134 (13)	0.0255 (5)	
H72	0.4830	0.1080	0.0640	0.031*	
C73	0.4845 (3)	−0.0111 (2)	0.07757 (15)	0.0324 (6)	
H73	0.5818	−0.0176	0.0740	0.039*	
C74	0.4020 (3)	−0.07579 (19)	0.08886 (13)	0.0299 (6)	
H74	0.4428	−0.1263	0.0937	0.036*	
C75	0.2597 (3)	−0.06688 (18)	0.09311 (13)	0.0283 (6)	
H75	0.2027	−0.1115	0.1004	0.034*	
C76	0.2004 (3)	0.00746 (17)	0.08673 (12)	0.0224 (5)	
H76	0.1028	0.0133	0.0899	0.027*	
C81	0.0388 (2)	0.17016 (15)	0.09452 (11)	0.0161 (4)	
C82	0.0243 (3)	0.17916 (18)	0.15620 (12)	0.0234 (5)	
H82	0.1054	0.1864	0.1840	0.028*	
C83	−0.1077 (3)	0.1775 (2)	0.17707 (13)	0.0307 (6)	
H83	−0.1167	0.1828	0.2189	0.037*	
C84	−0.2265 (3)	0.1683 (2)	0.13677 (14)	0.0336 (7)	
H84	−0.3169	0.1678	0.1510	0.040*	
C85	−0.2131 (3)	0.1599 (2)	0.07591 (14)	0.0320 (7)	
H85	−0.2947	0.1537	0.0485	0.038*	
C86	−0.0806 (3)	0.16040 (18)	0.05428 (12)	0.0238 (5)	
H86	−0.0723	0.1541	0.0123	0.029*	
C91	0.1855 (2)	0.16749 (15)	−0.00985 (10)	0.0146 (4)	
C92	0.1600 (2)	0.09072 (16)	−0.05353 (11)	0.0168 (4)	
H92	0.1673	0.0387	−0.0421	0.020*	
C93	0.1239 (3)	0.09019 (18)	−0.11384 (11)	0.0218 (5)	
H93	0.1083	0.0379	−0.1436	0.026*	
C94	0.1108 (3)	0.16631 (18)	−0.13052 (11)	0.0231 (5)	
H94	0.0826	0.1659	−0.1714	0.028*	
C95	0.1388 (3)	0.24292 (17)	−0.08762 (12)	0.0224 (5)	
H95	0.1314	0.2948	−0.0993	0.027*	
C96	0.1776 (3)	0.24370 (16)	−0.02761 (11)	0.0197 (5)	
H96	0.1989	0.2961	0.0015	0.024*	
O1	0.29782 (18)	0.28441 (11)	0.52369 (8)	0.0178 (3)	
O2	0.52335 (19)	0.30909 (13)	0.51366 (10)	0.0287 (4)	
O4	0.17335 (17)	0.40868 (11)	0.14454 (8)	0.0181 (3)	
O6	0.0685 (2)	0.45087 (16)	0.06758 (10)	0.0339 (5)	
O7	−0.1503 (2)	0.54379 (13)	0.04566 (9)	0.0258 (4)	
H7A	−0.088 (3)	0.5092 (18)	0.0496 (16)	0.031*	
H7B	−0.153 (4)	0.543 (2)	0.0086 (6)	0.031*	
O8	0.6376 (2)	0.17707 (13)	0.54856 (9)	0.0254 (4)	
H8C	0.591 (3)	0.2133 (17)	0.5363 (15)	0.030*	
H8D	0.699 (3)	0.172 (2)	0.5241 (12)	0.030*	
C10	0.2216 (3)	0.71407 (19)	0.10551 (14)	0.0289 (6)	
C20	0.1970 (4)	0.6458 (2)	0.05039 (15)	0.0396 (8)	
H20A	0.1073	0.6525	0.0288	0.059*	
H20B	0.1943	0.5911	0.0610	0.059*	
H20C	0.2728	0.6480	0.0243	0.059*	

### Atomic displacement parameters (Å^2^)

**Table:**

	*U*^11^	*U*^22^	*U*^33^	*U*^12^	*U*^13^	*U*^23^
Ag1	0.01534 (8)	0.01640 (8)	0.01295 (8)	−0.00237 (6)	−0.00027 (6)	0.00259 (6)
Ag2	0.01955 (9)	0.01800 (9)	0.01197 (8)	0.00333 (6)	0.00106 (6)	0.00327 (6)
S1	0.0137 (2)	0.0150 (3)	0.0165 (3)	−0.00022 (19)	0.00441 (19)	0.0032 (2)
S2	0.0175 (3)	0.0181 (3)	0.0138 (2)	−0.0006 (2)	−0.0003 (2)	0.0055 (2)
S3	0.0233 (3)	0.0220 (3)	0.0224 (3)	−0.0022 (2)	0.0107 (2)	0.0033 (2)
P1	0.0120 (2)	0.0129 (3)	0.0099 (2)	0.00055 (19)	0.00173 (19)	0.00199 (19)
P2	0.0120 (2)	0.0137 (3)	0.0110 (2)	−0.0001 (2)	0.00039 (19)	0.0031 (2)
P3	0.0127 (3)	0.0154 (3)	0.0118 (2)	−0.0029 (2)	0.0012 (2)	0.0020 (2)
N1	0.0195 (10)	0.0164 (10)	0.0210 (10)	0.0005 (8)	0.0085 (8)	0.0038 (8)
N2	0.0245 (11)	0.0150 (10)	0.0255 (11)	0.0020 (8)	0.0122 (9)	0.0045 (8)
N3	0.0115 (9)	0.0175 (10)	0.0316 (12)	0.0023 (7)	−0.0015 (8)	0.0007 (9)
N4	0.0117 (9)	0.0199 (11)	0.0378 (13)	0.0011 (8)	−0.0014 (9)	0.0051 (9)
N5	0.0183 (10)	0.0225 (11)	0.0388 (14)	−0.0011 (8)	0.0103 (9)	0.0081 (10)
N6	0.0159 (10)	0.0192 (10)	0.0301 (12)	−0.0022 (8)	0.0063 (8)	0.0046 (9)
C1	0.0126 (10)	0.0140 (10)	0.0142 (10)	−0.0011 (8)	0.0010 (8)	0.0016 (8)
C2	0.0204 (12)	0.0207 (12)	0.0191 (11)	−0.0028 (9)	0.0065 (9)	0.0042 (9)
C3	0.0200 (12)	0.0243 (13)	0.0225 (12)	0.0031 (9)	0.0106 (9)	0.0083 (10)
C4	0.0135 (10)	0.0167 (11)	0.0200 (11)	−0.0002 (8)	0.0024 (8)	0.0082 (9)
C5	0.0166 (12)	0.0214 (14)	0.060 (2)	0.0029 (10)	−0.0037 (13)	−0.0095 (13)
C6	0.0192 (12)	0.0206 (13)	0.0376 (15)	0.0052 (10)	0.0014 (11)	0.0032 (11)
C7	0.0157 (11)	0.0209 (12)	0.0225 (12)	−0.0014 (9)	0.0033 (9)	0.0092 (9)
N7	0.0502 (17)	0.0299 (14)	0.0330 (14)	0.0094 (12)	−0.0059 (12)	0.0074 (11)
C8	0.0243 (14)	0.0217 (13)	0.0487 (18)	−0.0023 (11)	0.0091 (12)	0.0094 (12)
C9	0.0216 (12)	0.0203 (12)	0.0329 (14)	0.0025 (10)	0.0040 (10)	0.0076 (11)
C11	0.0142 (10)	0.0136 (10)	0.0147 (10)	−0.0002 (8)	0.0034 (8)	0.0031 (8)
C12	0.0200 (11)	0.0167 (11)	0.0165 (11)	−0.0005 (9)	0.0032 (9)	0.0023 (9)
C13	0.0264 (13)	0.0185 (12)	0.0215 (12)	−0.0042 (10)	0.0077 (10)	−0.0021 (9)
C14	0.0273 (13)	0.0164 (12)	0.0351 (15)	0.0033 (10)	0.0168 (11)	0.0008 (10)
C15	0.0201 (12)	0.0205 (13)	0.0388 (16)	0.0064 (10)	0.0070 (11)	0.0072 (11)
C16	0.0172 (11)	0.0196 (12)	0.0232 (12)	0.0030 (9)	0.0013 (9)	0.0014 (9)
C21	0.0139 (10)	0.0128 (10)	0.0132 (10)	0.0005 (8)	0.0019 (8)	0.0013 (8)
C22	0.0174 (11)	0.0190 (11)	0.0166 (11)	0.0026 (9)	0.0016 (8)	0.0052 (9)
C23	0.0210 (12)	0.0243 (13)	0.0198 (12)	0.0059 (10)	−0.0027 (9)	0.0034 (10)
C24	0.0148 (11)	0.0221 (12)	0.0276 (13)	0.0020 (9)	−0.0006 (9)	−0.0010 (10)
C25	0.0155 (11)	0.0217 (12)	0.0246 (12)	−0.0030 (9)	0.0070 (9)	0.0011 (10)
C26	0.0160 (11)	0.0173 (11)	0.0173 (11)	0.0002 (8)	0.0039 (8)	0.0030 (9)
C31	0.0117 (9)	0.0152 (10)	0.0119 (9)	−0.0014 (8)	−0.0009 (7)	0.0035 (8)
C30	0.0147 (10)	0.0178 (11)	0.0203 (11)	−0.0007 (8)	−0.0004 (8)	0.0059 (9)
C32	0.0165 (11)	0.0212 (12)	0.0136 (10)	−0.0009 (9)	0.0026 (8)	0.0018 (9)
C33	0.0209 (12)	0.0299 (14)	0.0177 (11)	−0.0035 (10)	0.0045 (9)	0.0074 (10)
C34	0.0216 (12)	0.0262 (13)	0.0251 (13)	−0.0058 (10)	−0.0003 (10)	0.0127 (10)
C35	0.0236 (12)	0.0172 (11)	0.0256 (13)	−0.0007 (9)	0.0025 (10)	0.0074 (10)
C36	0.0187 (11)	0.0166 (11)	0.0165 (11)	0.0002 (9)	0.0040 (8)	0.0037 (9)
C41	0.0162 (10)	0.0154 (10)	0.0121 (10)	−0.0008 (8)	−0.0012 (8)	0.0049 (8)
C40	0.0271 (14)	0.0197 (13)	0.0434 (17)	−0.0021 (10)	0.0022 (12)	0.0143 (12)
C42	0.0168 (11)	0.0184 (11)	0.0212 (12)	−0.0023 (9)	0.0027 (9)	0.0043 (9)
C43	0.0172 (11)	0.0233 (13)	0.0299 (14)	−0.0054 (9)	0.0009 (10)	0.0093 (10)
C44	0.0254 (13)	0.0175 (12)	0.0308 (14)	−0.0034 (10)	−0.0065 (11)	0.0064 (10)
C45	0.0304 (14)	0.0160 (11)	0.0213 (12)	−0.0001 (10)	−0.0025 (10)	−0.0004 (9)
C46	0.0209 (12)	0.0199 (12)	0.0167 (11)	0.0019 (9)	0.0022 (9)	0.0023 (9)
C51	0.0137 (10)	0.0167 (10)	0.0130 (10)	0.0006 (8)	0.0032 (8)	0.0054 (8)
C50	0.0168 (11)	0.0163 (11)	0.0160 (10)	0.0029 (8)	0.0036 (8)	0.0047 (8)
C52	0.0169 (11)	0.0180 (11)	0.0168 (11)	0.0002 (8)	0.0032 (8)	0.0049 (9)
C53	0.0241 (12)	0.0178 (12)	0.0237 (12)	0.0023 (9)	0.0053 (10)	0.0051 (9)
C54	0.0200 (12)	0.0257 (13)	0.0245 (12)	0.0067 (10)	0.0070 (9)	0.0133 (10)
C55	0.0176 (11)	0.0305 (14)	0.0226 (12)	0.0019 (10)	−0.0022 (9)	0.0124 (10)
C56	0.0170 (11)	0.0210 (12)	0.0199 (11)	−0.0007 (9)	−0.0020 (9)	0.0054 (9)
C61	0.0127 (10)	0.0148 (10)	0.0131 (10)	−0.0015 (8)	0.0015 (8)	0.0022 (8)
C60	0.0142 (11)	0.0269 (13)	0.0304 (14)	0.0000 (9)	0.0018 (9)	0.0122 (11)
C62	0.0189 (11)	0.0210 (12)	0.0145 (10)	0.0020 (9)	0.0016 (8)	0.0062 (9)
C63	0.0240 (12)	0.0194 (11)	0.0159 (11)	−0.0015 (9)	0.0045 (9)	0.0069 (9)
C64	0.0177 (11)	0.0216 (12)	0.0200 (11)	−0.0020 (9)	0.0070 (9)	0.0031 (9)
C65	0.0165 (11)	0.0315 (14)	0.0214 (12)	0.0062 (10)	0.0049 (9)	0.0093 (10)
C66	0.0158 (11)	0.0267 (13)	0.0163 (11)	0.0034 (9)	0.0031 (8)	0.0096 (9)
C71	0.0153 (10)	0.0197 (11)	0.0120 (10)	−0.0001 (8)	0.0003 (8)	0.0031 (8)
C72	0.0166 (12)	0.0298 (14)	0.0309 (14)	0.0018 (10)	0.0053 (10)	0.0082 (11)
C73	0.0232 (14)	0.0375 (17)	0.0369 (16)	0.0122 (12)	0.0029 (12)	0.0085 (13)
C74	0.0380 (16)	0.0276 (14)	0.0237 (13)	0.0110 (12)	−0.0027 (11)	0.0059 (11)
C75	0.0359 (15)	0.0226 (13)	0.0267 (14)	0.0001 (11)	−0.0005 (11)	0.0080 (11)
C76	0.0211 (12)	0.0211 (12)	0.0245 (13)	−0.0007 (9)	0.0012 (10)	0.0050 (10)
C81	0.0155 (10)	0.0158 (11)	0.0163 (10)	−0.0026 (8)	0.0044 (8)	0.0024 (8)
C82	0.0228 (12)	0.0295 (14)	0.0182 (12)	−0.0027 (10)	0.0054 (9)	0.0058 (10)
C83	0.0321 (15)	0.0379 (16)	0.0224 (13)	−0.0029 (12)	0.0153 (11)	0.0046 (12)
C84	0.0222 (13)	0.0395 (17)	0.0350 (16)	−0.0066 (12)	0.0158 (12)	−0.0014 (13)
C85	0.0150 (12)	0.0482 (19)	0.0289 (14)	−0.0052 (12)	0.0057 (10)	0.0020 (13)
C86	0.0155 (11)	0.0340 (15)	0.0197 (12)	−0.0047 (10)	0.0042 (9)	0.0024 (10)
C91	0.0106 (9)	0.0182 (11)	0.0142 (10)	−0.0019 (8)	0.0015 (8)	0.0030 (8)
C92	0.0158 (10)	0.0185 (11)	0.0156 (10)	−0.0012 (8)	0.0038 (8)	0.0025 (8)
C93	0.0214 (12)	0.0270 (13)	0.0145 (11)	−0.0012 (10)	0.0035 (9)	0.0001 (9)
C94	0.0211 (12)	0.0337 (14)	0.0141 (11)	−0.0007 (10)	0.0019 (9)	0.0057 (10)
C95	0.0247 (13)	0.0245 (13)	0.0197 (12)	0.0006 (10)	0.0022 (9)	0.0088 (10)
C96	0.0209 (12)	0.0191 (12)	0.0188 (11)	−0.0010 (9)	0.0028 (9)	0.0039 (9)
O1	0.0140 (8)	0.0153 (8)	0.0240 (9)	−0.0006 (6)	0.0032 (6)	0.0045 (7)
O2	0.0113 (8)	0.0303 (11)	0.0466 (13)	0.0004 (7)	0.0039 (8)	0.0133 (9)
O4	0.0128 (8)	0.0234 (9)	0.0193 (8)	0.0024 (6)	0.0015 (6)	0.0075 (7)
O6	0.0216 (10)	0.0611 (15)	0.0306 (11)	0.0087 (10)	0.0066 (8)	0.0312 (11)
O7	0.0241 (10)	0.0294 (10)	0.0280 (10)	0.0050 (8)	0.0099 (8)	0.0124 (8)
O8	0.0221 (9)	0.0290 (10)	0.0298 (10)	0.0070 (8)	0.0111 (8)	0.0126 (8)
C10	0.0291 (14)	0.0287 (15)	0.0308 (15)	0.0032 (11)	0.0002 (11)	0.0117 (12)
C20	0.0369 (17)	0.0422 (19)	0.0332 (17)	−0.0070 (14)	0.0029 (13)	−0.0016 (14)

### Geometric parameters (Å, º)

**Table:**

Ag1—P3	2.4381 (6)	C34—H34	0.9500
Ag1—P2	2.4744 (6)	C35—C36	1.391 (3)
Ag1—S3	2.5424 (7)	C35—H35	0.9500
Ag1—O4	2.5564 (17)	C36—H36	0.9500
Ag2—P1	2.4431 (6)	C41—C46	1.394 (3)
Ag2—S2	2.5200 (6)	C41—C42	1.400 (3)
Ag2—S1	2.5810 (6)	C40—H40A	0.9800
Ag2—S1^i^	2.7883 (6)	C40—H40B	0.9800
S1—C1	1.715 (2)	C40—H40C	0.9800
S1—Ag2^i^	2.7883 (6)	C42—C43	1.395 (3)
S2—C4	1.708 (3)	C42—H42	0.9500
S3—C7	1.704 (3)	C43—C44	1.387 (4)
P1—C11	1.824 (2)	C43—H43	0.9500
P1—C21	1.826 (2)	C44—C45	1.380 (4)
P1—C31	1.827 (2)	C44—H44	0.9500
P2—C51	1.824 (2)	C45—C46	1.395 (4)
P2—C41	1.826 (2)	C45—H45	0.9500
P2—C61	1.832 (2)	C46—H46	0.9500
P3—C91	1.814 (2)	C51—C52	1.399 (3)
P3—C71	1.820 (3)	C51—C56	1.399 (3)
P3—C81	1.824 (2)	C50—O6	1.256 (3)
N1—C1	1.327 (3)	C50—O4	1.259 (3)
N1—C2	1.470 (3)	C50—C60	1.514 (3)
N1—H1	0.8800	C52—C53	1.390 (3)
N2—C1	1.327 (3)	C52—H52	0.9500
N2—C3	1.462 (3)	C53—C54	1.395 (4)
N2—H2	0.8800	C53—H53	0.9500
N3—C4	1.333 (3)	C54—C55	1.386 (4)
N3—C5	1.463 (3)	C54—H54	0.9500
N3—H3	0.8800	C55—C56	1.395 (4)
N4—C4	1.337 (3)	C55—H55	0.9500
N4—C6	1.460 (4)	C56—H56	0.9500
N4—H4	0.8800	C61—C66	1.399 (3)
N5—C7	1.343 (3)	C61—C62	1.399 (3)
N5—C8	1.462 (4)	C60—H60A	0.9800
N5—H5	0.8800	C60—H60B	0.9800
N6—C7	1.330 (3)	C60—H60C	0.9800
N6—C9	1.468 (3)	C62—C63	1.392 (3)
N6—H6	0.8800	C62—H62	0.9500
C2—C3	1.541 (4)	C63—C64	1.394 (4)
C2—H2A	0.9900	C63—H63	0.9500
C2—H2B	0.9900	C64—C65	1.391 (4)
C3—H3A	0.9900	C64—H64	0.9500
C3—H3B	0.9900	C65—C66	1.397 (3)
C5—C6	1.523 (4)	C65—H65	0.9500
C5—H5A	0.9900	C66—H66	0.9500
C5—H5B	0.9900	C71—C76	1.391 (3)
C6—H6A	0.9900	C71—C72	1.397 (3)
C6—H6B	0.9900	C72—C73	1.394 (4)
N7—C10	1.138 (4)	C72—H72	0.9500
C8—C9	1.533 (4)	C73—C74	1.382 (5)
C8—H8A	0.9900	C73—H73	0.9500
C8—H8B	0.9900	C74—C75	1.385 (4)
C9—H9A	0.9900	C74—H74	0.9500
C9—H9B	0.9900	C75—C76	1.391 (4)
C11—C16	1.394 (3)	C75—H75	0.9500
C11—C12	1.401 (3)	C76—H76	0.9500
C12—C13	1.398 (3)	C81—C86	1.391 (3)
C12—H12	0.9500	C81—C82	1.400 (3)
C13—C14	1.384 (4)	C82—C83	1.388 (4)
C13—H13	0.9500	C82—H82	0.9500
C14—C15	1.386 (4)	C83—C84	1.387 (5)
C14—H14	0.9500	C83—H83	0.9500
C15—C16	1.396 (4)	C84—C85	1.380 (4)
C15—H15	0.9500	C84—H84	0.9500
C16—H16	0.9500	C85—C86	1.398 (4)
C21—C22	1.395 (3)	C85—H85	0.9500
C21—C26	1.400 (3)	C86—H86	0.9500
C22—C23	1.396 (3)	C91—C92	1.397 (3)
C22—H22	0.9500	C91—C96	1.401 (3)
C23—C24	1.385 (4)	C92—C93	1.393 (3)
C23—H23	0.9500	C92—H92	0.9500
C24—C25	1.391 (4)	C93—C94	1.391 (4)
C24—H24	0.9500	C93—H93	0.9500
C25—C26	1.394 (3)	C94—C95	1.389 (4)
C25—H25	0.9500	C94—H94	0.9500
C26—H26	0.9500	C95—C96	1.390 (3)
C31—C32	1.396 (3)	C95—H95	0.9500
C31—C36	1.402 (3)	C96—H96	0.9500
C30—O2	1.255 (3)	O7—H7A	0.848 (10)
C30—O1	1.259 (3)	O7—H7B	0.844 (10)
C30—C40	1.520 (4)	O8—H8C	0.842 (10)
C32—C33	1.398 (4)	O8—H8D	0.837 (10)
C32—H32	0.9500	C10—C20	1.459 (4)
C33—C34	1.385 (4)	C20—H20A	0.9800
C33—H33	0.9500	C20—H20B	0.9800
C34—C35	1.390 (4)	C20—H20C	0.9800
			
P3—Ag1—P2	123.16 (2)	C34—C33—H33	119.9
P3—Ag1—S3	114.75 (2)	C32—C33—H33	119.9
P2—Ag1—S3	118.40 (2)	C33—C34—C35	120.2 (2)
P3—Ag1—O4	99.89 (4)	C33—C34—H34	119.9
P2—Ag1—O4	91.92 (4)	C35—C34—H34	119.9
S3—Ag1—O4	97.56 (4)	C34—C35—C36	119.9 (2)
P1—Ag2—S2	130.88 (2)	C34—C35—H35	120.1
P1—Ag2—S1	100.98 (2)	C36—C35—H35	120.1
S2—Ag2—S1	122.99 (2)	C35—C36—C31	120.4 (2)
P1—Ag2—S1^i^	112.706 (19)	C35—C36—H36	119.8
S2—Ag2—S1^i^	92.547 (19)	C31—C36—H36	119.8
S1—Ag2—S1^i^	86.195 (19)	C46—C41—C42	119.1 (2)
C1—S1—Ag2	113.31 (8)	C46—C41—P2	123.41 (19)
C1—S1—Ag2^i^	117.47 (8)	C42—C41—P2	117.39 (18)
Ag2—S1—Ag2^i^	93.806 (19)	C30—C40—H40A	109.5
C4—S2—Ag2	103.55 (8)	C30—C40—H40B	109.5
C7—S3—Ag1	101.86 (9)	H40A—C40—H40B	109.5
C11—P1—C21	103.14 (10)	C30—C40—H40C	109.5
C11—P1—C31	103.96 (10)	H40A—C40—H40C	109.5
C21—P1—C31	103.77 (10)	H40B—C40—H40C	109.5
C11—P1—Ag2	119.00 (8)	C43—C42—C41	119.9 (2)
C21—P1—Ag2	114.48 (8)	C43—C42—H42	120.0
C31—P1—Ag2	110.90 (7)	C41—C42—H42	120.0
C51—P2—C41	104.65 (11)	C44—C43—C42	120.5 (2)
C51—P2—C61	102.56 (10)	C44—C43—H43	119.8
C41—P2—C61	104.66 (11)	C42—C43—H43	119.8
C51—P2—Ag1	113.13 (8)	C45—C44—C43	119.7 (2)
C41—P2—Ag1	111.70 (7)	C45—C44—H44	120.1
C61—P2—Ag1	118.76 (7)	C43—C44—H44	120.1
C91—P3—C71	105.22 (11)	C44—C45—C46	120.4 (2)
C91—P3—C81	103.32 (11)	C44—C45—H45	119.8
C71—P3—C81	103.63 (11)	C46—C45—H45	119.8
C91—P3—Ag1	116.27 (8)	C41—C46—C45	120.4 (2)
C71—P3—Ag1	114.34 (8)	C41—C46—H46	119.8
C81—P3—Ag1	112.68 (8)	C45—C46—H46	119.8
C1—N1—C2	111.9 (2)	C52—C51—C56	118.8 (2)
C1—N1—H1	124.0	C52—C51—P2	117.31 (17)
C2—N1—H1	124.0	C56—C51—P2	123.89 (19)
C1—N2—C3	111.8 (2)	O6—C50—O4	124.2 (2)
C1—N2—H2	124.1	O6—C50—C60	118.0 (2)
C3—N2—H2	124.1	O4—C50—C60	117.7 (2)
C4—N3—C5	111.5 (2)	C53—C52—C51	121.0 (2)
C4—N3—H3	124.3	C53—C52—H52	119.5
C5—N3—H3	124.3	C51—C52—H52	119.5
C4—N4—C6	111.2 (2)	C52—C53—C54	119.6 (2)
C4—N4—H4	124.4	C52—C53—H53	120.2
C6—N4—H4	124.4	C54—C53—H53	120.2
C7—N5—C8	110.7 (2)	C55—C54—C53	120.0 (2)
C7—N5—H5	124.6	C55—C54—H54	120.0
C8—N5—H5	124.6	C53—C54—H54	120.0
C7—N6—C9	111.3 (2)	C54—C55—C56	120.5 (2)
C7—N6—H6	124.4	C54—C55—H55	119.8
C9—N6—H6	124.4	C56—C55—H55	119.8
N1—C1—N2	110.7 (2)	C55—C56—C51	120.2 (2)
N1—C1—S1	125.64 (18)	C55—C56—H56	119.9
N2—C1—S1	123.62 (18)	C51—C56—H56	119.9
N1—C2—C3	102.27 (19)	C66—C61—C62	119.4 (2)
N1—C2—H2A	111.3	C66—C61—P2	117.91 (17)
C3—C2—H2A	111.3	C62—C61—P2	122.62 (18)
N1—C2—H2B	111.3	C50—C60—H60A	109.5
C3—C2—H2B	111.3	C50—C60—H60B	109.5
H2A—C2—H2B	109.2	H60A—C60—H60B	109.5
N2—C3—C2	102.88 (19)	C50—C60—H60C	109.5
N2—C3—H3A	111.2	H60A—C60—H60C	109.5
C2—C3—H3A	111.2	H60B—C60—H60C	109.5
N2—C3—H3B	111.2	C63—C62—C61	120.4 (2)
C2—C3—H3B	111.2	C63—C62—H62	119.8
H3A—C3—H3B	109.1	C61—C62—H62	119.8
N3—C4—N4	110.2 (2)	C62—C63—C64	120.1 (2)
N3—C4—S2	124.90 (19)	C62—C63—H63	120.0
N4—C4—S2	124.93 (19)	C64—C63—H63	120.0
N3—C5—C6	102.7 (2)	C65—C64—C63	119.9 (2)
N3—C5—H5A	111.2	C65—C64—H64	120.1
C6—C5—H5A	111.2	C63—C64—H64	120.1
N3—C5—H5B	111.2	C64—C65—C66	120.4 (2)
C6—C5—H5B	111.2	C64—C65—H65	119.8
H5A—C5—H5B	109.1	C66—C65—H65	119.8
N4—C6—C5	102.9 (2)	C65—C66—C61	119.9 (2)
N4—C6—H6A	111.2	C65—C66—H66	120.1
C5—C6—H6A	111.2	C61—C66—H66	120.1
N4—C6—H6B	111.2	C76—C71—C72	118.8 (2)
C5—C6—H6B	111.2	C76—C71—P3	123.77 (19)
H6A—C6—H6B	109.1	C72—C71—P3	117.33 (19)
N6—C7—N5	109.7 (2)	C73—C72—C71	120.2 (3)
N6—C7—S3	125.12 (19)	C73—C72—H72	119.9
N5—C7—S3	125.2 (2)	C71—C72—H72	119.9
N5—C8—C9	101.7 (2)	C74—C73—C72	120.3 (3)
N5—C8—H8A	111.4	C74—C73—H73	119.8
C9—C8—H8A	111.4	C72—C73—H73	119.8
N5—C8—H8B	111.4	C73—C74—C75	119.9 (3)
C9—C8—H8B	111.4	C73—C74—H74	120.1
H8A—C8—H8B	109.3	C75—C74—H74	120.1
N6—C9—C8	101.2 (2)	C74—C75—C76	120.0 (3)
N6—C9—H9A	111.5	C74—C75—H75	120.0
C8—C9—H9A	111.5	C76—C75—H75	120.0
N6—C9—H9B	111.5	C71—C76—C75	120.8 (3)
C8—C9—H9B	111.5	C71—C76—H76	119.6
H9A—C9—H9B	109.4	C75—C76—H76	119.6
C16—C11—C12	119.0 (2)	C86—C81—C82	119.3 (2)
C16—C11—P1	117.51 (18)	C86—C81—P3	122.70 (18)
C12—C11—P1	123.49 (18)	C82—C81—P3	118.02 (19)
C13—C12—C11	120.6 (2)	C83—C82—C81	120.5 (3)
C13—C12—H12	119.7	C83—C82—H82	119.7
C11—C12—H12	119.7	C81—C82—H82	119.7
C14—C13—C12	119.7 (2)	C84—C83—C82	119.9 (3)
C14—C13—H13	120.2	C84—C83—H83	120.0
C12—C13—H13	120.2	C82—C83—H83	120.0
C13—C14—C15	120.1 (2)	C85—C84—C83	119.9 (3)
C13—C14—H14	120.0	C85—C84—H84	120.0
C15—C14—H14	120.0	C83—C84—H84	120.0
C14—C15—C16	120.5 (3)	C84—C85—C86	120.6 (3)
C14—C15—H15	119.7	C84—C85—H85	119.7
C16—C15—H15	119.7	C86—C85—H85	119.7
C11—C16—C15	120.0 (2)	C81—C86—C85	119.7 (2)
C11—C16—H16	120.0	C81—C86—H86	120.1
C15—C16—H16	120.0	C85—C86—H86	120.1
C22—C21—C26	119.6 (2)	C92—C91—C96	119.3 (2)
C22—C21—P1	122.98 (18)	C92—C91—P3	123.29 (18)
C26—C21—P1	117.27 (17)	C96—C91—P3	117.23 (18)
C21—C22—C23	120.0 (2)	C93—C92—C91	120.2 (2)
C21—C22—H22	120.0	C93—C92—H92	119.9
C23—C22—H22	120.0	C91—C92—H92	119.9
C24—C23—C22	120.4 (2)	C94—C93—C92	120.0 (2)
C24—C23—H23	119.8	C94—C93—H93	120.0
C22—C23—H23	119.8	C92—C93—H93	120.0
C23—C24—C25	119.7 (2)	C95—C94—C93	120.2 (2)
C23—C24—H24	120.2	C95—C94—H94	119.9
C25—C24—H24	120.2	C93—C94—H94	119.9
C24—C25—C26	120.6 (2)	C94—C95—C96	120.0 (2)
C24—C25—H25	119.7	C94—C95—H95	120.0
C26—C25—H25	119.7	C96—C95—H95	120.0
C25—C26—C21	119.6 (2)	C95—C96—C91	120.3 (2)
C25—C26—H26	120.2	C95—C96—H96	119.9
C21—C26—H26	120.2	C91—C96—H96	119.9
C32—C31—C36	119.2 (2)	C50—O4—Ag1	132.75 (16)
C32—C31—P1	123.68 (18)	H7A—O7—H7B	104 (3)
C36—C31—P1	117.06 (17)	H8C—O8—H8D	99 (3)
O2—C30—O1	124.5 (2)	N7—C10—C20	178.5 (3)
O2—C30—C40	117.0 (2)	C10—C20—H20A	109.5
O1—C30—C40	118.5 (2)	C10—C20—H20B	109.5
C31—C32—C33	120.0 (2)	H20A—C20—H20B	109.5
C31—C32—H32	120.0	C10—C20—H20C	109.5
C33—C32—H32	120.0	H20A—C20—H20C	109.5
C34—C33—C32	120.2 (2)	H20B—C20—H20C	109.5
			
C2—N1—C1—N2	−0.9 (3)	C41—C42—C43—C44	−1.8 (4)
C2—N1—C1—S1	177.63 (18)	C42—C43—C44—C45	0.6 (4)
C3—N2—C1—N1	5.0 (3)	C43—C44—C45—C46	1.0 (4)
C3—N2—C1—S1	−173.59 (18)	C42—C41—C46—C45	0.1 (4)
Ag2—S1—C1—N1	22.0 (2)	P2—C41—C46—C45	−175.3 (2)
Ag2^i^—S1—C1—N1	129.75 (19)	C44—C45—C46—C41	−1.3 (4)
Ag2—S1—C1—N2	−159.64 (18)	C41—P2—C51—C52	−164.80 (18)
Ag2^i^—S1—C1—N2	−51.9 (2)	C61—P2—C51—C52	86.16 (19)
C1—N1—C2—C3	−3.1 (3)	Ag1—P2—C51—C52	−43.0 (2)
C1—N2—C3—C2	−6.6 (3)	C41—P2—C51—C56	17.0 (2)
N1—C2—C3—N2	5.5 (3)	C61—P2—C51—C56	−92.1 (2)
C5—N3—C4—N4	−2.1 (3)	Ag1—P2—C51—C56	138.78 (19)
C5—N3—C4—S2	176.5 (2)	C56—C51—C52—C53	0.2 (4)
C6—N4—C4—N3	−6.6 (3)	P2—C51—C52—C53	−178.08 (19)
C6—N4—C4—S2	174.8 (2)	C51—C52—C53—C54	−0.3 (4)
Ag2—S2—C4—N3	54.9 (2)	C52—C53—C54—C55	0.1 (4)
Ag2—S2—C4—N4	−126.7 (2)	C53—C54—C55—C56	0.1 (4)
C4—N3—C5—C6	9.1 (4)	C54—C55—C56—C51	−0.1 (4)
C4—N4—C6—C5	11.8 (3)	C52—C51—C56—C55	0.0 (4)
N3—C5—C6—N4	−11.9 (3)	P2—C51—C56—C55	178.18 (19)
C9—N6—C7—N5	−7.6 (3)	C51—P2—C61—C66	−156.93 (19)
C9—N6—C7—S3	171.8 (2)	C41—P2—C61—C66	94.0 (2)
C8—N5—C7—N6	−8.6 (3)	Ag1—P2—C61—C66	−31.4 (2)
C8—N5—C7—S3	172.0 (2)	C51—P2—C61—C62	21.0 (2)
Ag1—S3—C7—N6	−23.8 (2)	C41—P2—C61—C62	−88.0 (2)
Ag1—S3—C7—N5	155.5 (2)	Ag1—P2—C61—C62	146.59 (17)
C7—N5—C8—C9	19.8 (3)	C66—C61—C62—C63	0.5 (4)
C7—N6—C9—C8	19.2 (3)	P2—C61—C62—C63	−177.46 (19)
N5—C8—C9—N6	−22.1 (3)	C61—C62—C63—C64	−0.4 (4)
C21—P1—C11—C16	160.75 (19)	C62—C63—C64—C65	−0.3 (4)
C31—P1—C11—C16	−91.2 (2)	C63—C64—C65—C66	1.0 (4)
Ag2—P1—C11—C16	32.7 (2)	C64—C65—C66—C61	−0.9 (4)
C21—P1—C11—C12	−20.0 (2)	C62—C61—C66—C65	0.2 (4)
C31—P1—C11—C12	88.0 (2)	P2—C61—C66—C65	178.2 (2)
Ag2—P1—C11—C12	−148.05 (18)	C91—P3—C71—C76	−99.6 (2)
C16—C11—C12—C13	0.0 (4)	C81—P3—C71—C76	8.6 (2)
P1—C11—C12—C13	−179.19 (19)	Ag1—P3—C71—C76	131.61 (19)
C11—C12—C13—C14	−0.3 (4)	C91—P3—C71—C72	83.7 (2)
C12—C13—C14—C15	0.8 (4)	C81—P3—C71—C72	−168.2 (2)
C13—C14—C15—C16	−1.1 (4)	Ag1—P3—C71—C72	−45.1 (2)
C12—C11—C16—C15	−0.3 (4)	C76—C71—C72—C73	0.2 (4)
P1—C11—C16—C15	178.9 (2)	P3—C71—C72—C73	177.2 (2)
C14—C15—C16—C11	0.9 (4)	C71—C72—C73—C74	−0.7 (5)
C11—P1—C21—C22	82.9 (2)	C72—C73—C74—C75	1.0 (5)
C31—P1—C21—C22	−25.3 (2)	C73—C74—C75—C76	−0.7 (4)
Ag2—P1—C21—C22	−146.29 (18)	C72—C71—C76—C75	0.0 (4)
C11—P1—C21—C26	−92.77 (19)	P3—C71—C76—C75	−176.8 (2)
C31—P1—C21—C26	159.03 (18)	C74—C75—C76—C71	0.3 (4)
Ag2—P1—C21—C26	38.0 (2)	C91—P3—C81—C86	1.9 (2)
C26—C21—C22—C23	3.3 (4)	C71—P3—C81—C86	−107.7 (2)
P1—C21—C22—C23	−172.32 (19)	Ag1—P3—C81—C86	128.2 (2)
C21—C22—C23—C24	−1.5 (4)	C91—P3—C81—C82	−176.4 (2)
C22—C23—C24—C25	−1.0 (4)	C71—P3—C81—C82	74.0 (2)
C23—C24—C25—C26	1.8 (4)	Ag1—P3—C81—C82	−50.1 (2)
C24—C25—C26—C21	−0.1 (4)	C86—C81—C82—C83	0.6 (4)
C22—C21—C26—C25	−2.4 (4)	P3—C81—C82—C83	179.0 (2)
P1—C21—C26—C25	173.39 (19)	C81—C82—C83—C84	−1.0 (5)
C11—P1—C31—C32	−0.4 (2)	C82—C83—C84—C85	0.6 (5)
C21—P1—C31—C32	107.2 (2)	C83—C84—C85—C86	0.2 (5)
Ag2—P1—C31—C32	−129.43 (18)	C82—C81—C86—C85	0.1 (4)
C11—P1—C31—C36	177.69 (18)	P3—C81—C86—C85	−178.1 (2)
C21—P1—C31—C36	−74.73 (19)	C84—C85—C86—C81	−0.5 (5)
Ag2—P1—C31—C36	48.66 (19)	C71—P3—C91—C92	25.0 (2)
C36—C31—C32—C33	0.2 (3)	C81—P3—C91—C92	−83.4 (2)
P1—C31—C32—C33	178.25 (19)	Ag1—P3—C91—C92	152.64 (17)
C31—C32—C33—C34	0.2 (4)	C71—P3—C91—C96	−159.91 (19)
C32—C33—C34—C35	−0.2 (4)	C81—P3—C91—C96	91.7 (2)
C33—C34—C35—C36	−0.2 (4)	Ag1—P3—C91—C96	−32.3 (2)
C34—C35—C36—C31	0.6 (4)	C96—C91—C92—C93	−1.5 (3)
C32—C31—C36—C35	−0.6 (4)	P3—C91—C92—C93	173.51 (19)
P1—C31—C36—C35	−178.78 (19)	C91—C92—C93—C94	−1.1 (4)
C51—P2—C41—C46	−115.2 (2)	C92—C93—C94—C95	2.5 (4)
C61—P2—C41—C46	−7.7 (2)	C93—C94—C95—C96	−1.1 (4)
Ag1—P2—C41—C46	122.04 (19)	C94—C95—C96—C91	−1.5 (4)
C51—P2—C41—C42	69.4 (2)	C92—C91—C96—C95	2.8 (4)
C61—P2—C41—C42	176.87 (19)	P3—C91—C96—C95	−172.5 (2)
Ag1—P2—C41—C42	−53.4 (2)	O6—C50—O4—Ag1	−80.3 (3)
C46—C41—C42—C43	1.4 (4)	C60—C50—O4—Ag1	101.4 (2)
P2—C41—C42—C43	177.1 (2)		

Symmetry code: (i) −*x*, −*y*, −*z*+1.

### Hydrogen-bond geometry (Å, º)

**Table:**

*D*—H···*A*	*D*—H	H···*A*	*D*···*A*	*D*—H···*A*
N1—H1···O1	0.88	2.10	2.908 (3)	153
N2—H2···O8^ii^	0.88	2.00	2.808 (3)	152
N3—H3···O1	0.88	1.94	2.780 (3)	160
N4—H4···O2^iii^	0.88	2.09	2.864 (3)	146
N5—H5···O7^iv^	0.88	1.96	2.767 (3)	152
N6—H6···O4	0.88	1.91	2.780 (3)	172
O7—H7*A*···O6	0.85 (1)	1.88 (1)	2.716 (3)	169 (4)
O7—H7*B*···O6^v^	0.84 (1)	2.00 (2)	2.782 (3)	154 (3)
O8—H8*C*···O2	0.84 (1)	1.88 (1)	2.709 (3)	167 (3)
O8—H8*D*···S2^iv^	0.84 (1)	2.56 (2)	3.357 (2)	159 (3)

Symmetry codes: (ii) −*x*+1, −*y*, −*z*+1; (iii) *x*−1, *y*, *z*; (iv) *x*+1, *y*, *z*; (v) −*x*, −*y*+1, −*z*.
